# Optical Coherence Tomography and Angiography in Hydroxychloroquine Retinopathy: A Narrative Review

**DOI:** 10.3390/diagnostics16030463

**Published:** 2026-02-02

**Authors:** Alexandra Lori Donica, Vlad Constantin Donica, Mara Russu, Vladia Lăpuște, Cristina Pomîrleanu, Camelia Margareta Bogdănici, Anisia Iuliana Alexa, Călina Anda Sandu, Ioana Mădălina Bilha, Codrina Ancuța

**Affiliations:** 1 Grigore T. Popa University of Medicine and Pharmacy, University Street, No. 16, 700115 Iasi, Romania; costachescu.alexandra-lori@d.umfiasi.ro (A.L.D.); camelia.bogdanici@umfiasi.ro (C.M.B.); codrina.ancuta@umfiasi.ro (C.A.); 2 2nd Rheumatology Department, Clinical Rehabilitation Hospital, 700661 Iasi, Romania

**Keywords:** hydroxychloroquine, optical coherence tomography, optical coherence tomography-angiography, toxic maculopathy, bull’s eye maculopathy, retinal toxicity

## Abstract

**Background/Objectives**: Hydroxychloroquine (HCQ) is widely used in the treatment of autoimmune rheumatologic diseases due to its immunomodulatory and anti-inflammatory properties. However, long-term HCQ therapy carries a risk of irreversible retinal toxicity caused by drug accumulation in the retinal pigment epithelium. The early identification of preclinical retinal changes is essential to prevent permanent visual impairment. Optical coherence tomography (OCT) and OCT-angiography (OCT-A) have emerged as key imaging modalities for the detection of structural and microvascular biomarkers of HCQ retinopathy. A narrative review of the literature was conducted using the PubMed database, focusing on studies published between January 2017 and February 2025. Search terms included “hydroxychloroquine” and “optical coherence tomography.” Eligible studies evaluated HCQ-related retinal toxicity using OCT and/or OCT-A in human subjects. Data were extracted regarding study population characteristics, treatment duration, cumulative HCQ dose, daily dose normalized to real body weight, and reported imaging findings. **Results**: We identified 223 scientific papers of which 88 studies met the inclusion criteria. Structural OCT parameters—particularly alterations in the ellipsoid zone, outer nuclear layer, and retinal pigment epithelium—were consistently associated with early HCQ toxicity, often preceding functional impairment. OCT-A studies demonstrated microvascular alterations, including reduced vessel density and foveal avascular zone enlargement, though interpretation may be confounded by underlying autoimmune-disease-related vasculopathy. **Conclusions**: HCQ retinopathy is a potentially vision-threatening condition associated with the cumulative dose, treatment duration, and patient-specific risk factors. OCT and OCT-A provide complementary structural and vascular biomarkers that aid in the detection of subclinical retinal toxicity. The integration of quantitative and automated OCT-derived metrics may improve screening strategies, facilitate early diagnosis, and support personalized care in patients receiving long-term HCQ therapy.

## 1. Introduction

Hydroxychloroquine (HCQ) is a synthetic antimalarial drug used in the management of autoimmune rheumatologic diseases such as rheumatoid arthritis (RA), systemic lupus erythematosus (SLE), and Sjögren’s syndrome (SJS) due to its well-established immunomodulatory properties and anti-inflammatory effects [1]. By accumulating in lysosomes and autophagosomes, it increases the pH, resulting in the inhibition of the major histocompatibility complex class-II antigen presentation and subsequent activation of T lymphocytes, affecting both the innate and adaptive immune responses. The accumulation of HCQ blocks viral recognition by endosomal toll-like receptors, leading to a reduction in the innate antiviral response (decreased interferon-1 production). HCQ directly affects the adaptive immune system by inhibiting the differentiation and activation of T- and B-cell lymphocytes [2].

In addition to its immunomodulatory functions, HCQ also exerts additional pleiotropic effects including anti-inflammatory, antithrombotic, hypoglycemic, hypolipidemic, anticholinesterase, and photoprotective effects. Although initially proposed as a suppressive treatment for malaria, HCQ today plays an important role in modulating the activity of chronic inflammatory diseases, both as a monotherapy, by reducing disease activity and improving joint status, and in combination with other therapeutic classes, enhancing the overall favorable prognosis. The efficacy of HCQ in RA has been demonstrated both as a monotherapy in mild disease and, more importantly, in combination with other synthetic or biologic disease-modifying antirheumatic drugs in severe forms, in line with EULAR recommendations. In SLE, HCQ is recommended across all disease phenotypes, where it plays a crucial role in long-term disease modulation. Its hypolipidemic effect confers additional cardiovascular protection in RA/SLE patients with concomitant cardiovascular involvement. Recent studies further suggest that HCQ may be indicated in RA with chronic kidney disease, showing no significant impact on creatinine clearance, and may serve as an adjuvant in remission-induction therapy for lupus nephritis [3,4].

Nevertheless, the therapeutic benefits of HCQ must be balanced against its potential adverse effects. Prolonged HCQ exposure carries a well-recognized risk of retinal toxicity affecting the retina, ciliary body, and cornea, manifesting as bull’s-eye maculopathy, color vision disturbances, and paracentral scotomas, which may progress even after treatment discontinuation and can result in irreversible visual impairment. Accordingly, the discontinuation of therapy is warranted upon the onset of pathological ocular changes.

HCQ-induced retinal toxicity is primarily attributed to drug accumulation within melanin rich ocular tissues, particularly the retinal pigment epithelium (RPE). The high lipophilicity of HCQ, its large volume of distribution, and prolonged half-life facilitate intracellular accumulation within lysosomes, where the disruption of autophagic and phagolysosomal pathways in RPE cells impairs photoreceptor (PR) outer segment phagocytosis. This process is further compounded by the inhibition of all-trans retinol recycling via organic-anion-transporting polypeptide 1A2, resulting in early PR dysfunction. These molecular alterations manifest as the parafoveal attenuation or focal disruption of the ellipsoid zone (EZ), reflecting the early compromise of the PR inner segment integrity, followed by the progressive thinning of the outer nuclear layer (ONL), which represents PR cell body loss and is among the earliest quantitative biomarkers of HCQ toxicity. Autophagy-lysosomal defects and an altered sphingolipid metabolism may also contribute to the early involvement of inner retinal (IR) neurons, including retinal ganglion cells (RGC), providing a mechanistic substrate for the subtle IR thinning detected on Optical Coherence Tomography (OCT) in some cohorts. Additional pathways, such as TRPM2-mediated Ca^2+^ influx and oxidative retinal injury, likely accelerate PR and RPE degeneration, reinforcing the progressive structural changes observed on longitudinal OCT imaging. An additional consideration in minimizing the risk of retinal toxicity is to avoid the concomitant use of HCQ with other agents known to cause ocular damage [5,6]. Given these metabolic alterations, secondary vascular involvement could affect the perfusion of the retinal vascular layers, offering a potential explanation to the preferential toxicity involvement. However, these vascular findings need to be interpreted with caution as systemic autoimmune diseases may lead to choroidal thickness alterations and inflammatory microvascular remodeling in the absence of HCQ involvement.

A critical challenge in HCQ retinopathy is that early structural damage often pre-cedes visual symptoms, rendering the traditional ophthalmic examination insufficient for a timely diagnosis. Consequently, modern screening strategies increasingly rely on advanced retinal imaging modalities capable of detecting subclinical changes before functional loss occurs. The early detection of retinal toxicity is possible through Optical Coherence Tomography (OCT), multifocal electroretinography (mfERG), and fundus autofluorescence (FAF) [7].

### Optical Coherence Tomography

OCT is a non-invasive imaging modality based on low-coherence interferometry, widely applied in ophthalmology to generate high-resolution cross-sectional images of retinal layers. It enables a quantitative and qualitative assessment of key structures such as the retinal nerve fiber layer (RNFL), ganglion cell layer (GCL), macular thickness (MT) and architecture, and optic nerve head [8].

In the context of HCQ toxicity, OCT plays a pivotal role in the early identification of subclinical retinal alterations, before the onset of visual symptoms. The characteristic OCT findings associated with HCQ toxicity include the parafoveal disruption of the EZ, RPE degeneration, the thinning of the ONL, and changes in the inner plexiform layer (IPL), all of which represent early structural hallmarks of drug-induced retinal damage [7].

Although retinal thickness (RT) typically remains stable for many years in patients undergoing long-term HCQ therapy, evidence suggests that, beyond a critical cumulative exposure, the retina may begin to thin rapidly [9]. A quantitative analysis of MT across the inner and outer ETDRS rings can reveal these subtle structural alterations several years before the conventional clinical signs of toxicity become apparent. Sequential OCT monitoring therefore provides an objective and sensitive method for detecting early retinal involvement, enabling timely intervention and the adjustment of HCQ therapy. Because these measurements are derived from standard, widely available OCT protocols, their automation and integration into routine ophthalmic practice could substantially enhance the early detection of HCQ-induced retinal damage and improve long-term visual outcomes [10].

The existing literature on HCQ retinopathy assessed by OCT and OCT-A is characterized by considerable heterogeneity, encompassing cross-sectional and retrospective studies, variable imaging protocols, inconsistent segmentation methodologies, and an expanding body of exploratory research on early structural and vascular biomarkers. This methodological fragmentation limits the feasibility of systematic synthesis or meta-analytic comparison. In this context, a narrative review represents an appropriate framework to integrate diverse forms of evidence, contextualize emerging imaging biomarkers, and synthesize current knowledge across structural and vascular parameters. Accordingly, the aim of this review is to synthesize available evidence on OCT- and OCT-A-derived retinal alterations in patients receiving HCQ. Specifically, this review seeks to identify early OCT biomarkers associated with HCQ-induced retinal toxicity, characterize sequential patterns of structural retinal involvement, and evaluate the emerging role of OCT-A in detecting microvascular changes and its complementary value to structural OCT. By integrating data on outer retinal (OR), inner retinal (IR), choroidal, and microvascular changes, this review seeks to clarify the role of OCT-based biomarkers in the early detection, risk stratification, and longitudinal monitoring of HCQ retinopathy, thereby supporting optimized screening strategies and personalized patient care.

## 2. Materials and Methods

A search was conducted in the PubMed database to identify relevant studies published between January 2017 and February 2025. The search strategy combined free-text keywords and Medical Subject Headings (MeSH), including “hydroxychloroquine,” “chloroquine,” “retinal toxicity,” “maculopathy,” “optical coherence tomography,” “OCT,” “OCT angiography,” and “optical coherence tomography angiography,” using Boolean operators (AND/OR) to maximize sensitivity. Reference lists of all included articles and relevant reviews were manually screened to identify additional pertinent studies not captured by the initial search.

Studies were considered eligible if they met the following criteria: they included human subjects receiving HCQ therapy; evaluated retinal toxicity or structural and/or vascular retinal changes using (OCT) and/or OCT-A; and reported qualitative and/or quantitative imaging findings relevant to HCQ exposure. Observational study designs were included, such as cross-sectional, cohort, case–control, and retrospective analyses. Studies were excluded if they were limited to isolated case reports or small case series without systematic imaging-based analysis; focused exclusively on non-OCT imaging modalities without OCT or OCT-A correlation; involved experimental animal models or in vitro data; or were not available in the English language.

Titles and abstracts were initially screened for relevance, followed by full-text review of potentially eligible studies. Duplicate records were removed prior to full-text assessment. Study selection and data extraction were performed by the authors through consensus-based review, with discrepancies resolved by discussion.

Data extraction was performed using a predefined framework to ensure consistency across heterogeneous study designs. Imaging findings were collected as reported in the original studies. Structural OCT outcomes were grouped into OR parameters (including EZ integrity and ONL thickness), IR metrics (such as GCL and IPL measurements), and choroidal parameters, while OCT-A outcomes were categorized by vascular plexus involvement and reported quantitative or qualitative perfusion metrics.

Treatment-related variables, including duration of HCQ exposure, cumulative dose, and daily dose normalized to body weight, were extracted when available and interpreted in relative rather than absolute terms, acknowledging variability in reporting thresholds across studies. When quantitative values were not directly comparable due to differences in imaging platforms, segmentation algorithms, or acquisition protocols, findings were synthesized qualitatively based on directional consistency (thinning, disruption, and reduced vessel density) and anatomical localization.

Given the substantial heterogeneity in study design, patient populations, imaging platforms, and outcome reporting, a structured narrative synthesis approach was employed. Rather than pooling results quantitatively, studies were compared and integrated based on shared biological and anatomical targets affected by hydroxychloroquine toxicity. Grouping findings by anatomical domain allowed identification of convergent patterns across diverse methodologies while minimizing bias introduced by platform-specific measurements. Differences related to underlying autoimmune disease, imaging technology, and study design were considered during interpretation and explicitly discussed as potential sources of variability. Findings were grouped according to the primary anatomical region or imaging domain assessed, including OR, IR, EZ, choroid, and retinal microvasculature.

## 3. Results

**Figure 1:**
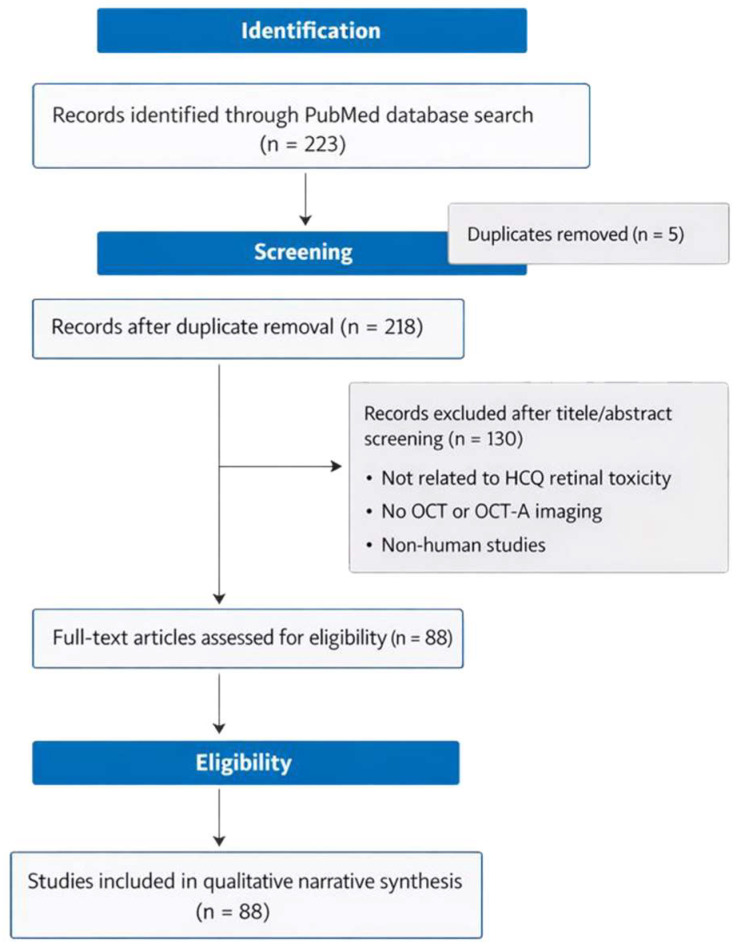
Flow diagram of study selection.

The search identified a total of 223 records through the PubMed database. After the removal of 5 duplicate records, 218 unique articles remained and were screened based on the title and abstract. Of these, 130 records were excluded because they were not directly related to HCQ retinal toxicity, did not include OCT or OCT-angiography-based imaging, or involved non-human studies. The remaining 88 articles underwent a full-text assessment for eligibility and met the predefined inclusion criteria. These studies were subsequently included in the qualitative narrative synthesis and formed the basis of the present review. The study selection process is summarized in Figure 1.

### 3.1. Outer Retinal Alterations

Given the HCQ mechanism of action, the OR has been the main target in HCQ retinopathy screening, with OCT being able to provide data regarding thickness measurements in different macular areas, while more subjective analyses were required to identify lesions such as drusen-like deposits (DLDs), EZ hyporeflectivity, or PR loss.

Multiple studies have identified a thinner OR thickness that is correlated with the HCQ dose and treatment duration, with HCQ retinopathy progressing under a certain pattern, the inferior macular quadrant being frequently the most affected (Table 1).

Across the analyzed studies, there is a strong agreement that HCQ exposure is associated with early structural alterations in the OR, even in the absence of clinically apparent retinopathy. The most consistently reported finding is the parafoveal and perifoveal thinning of the ONL and ORT, observed across diverse cohorts and imaging platforms [9,12,15,16,20,23,30].

A preferential topographic pattern was repeatedly observed, with the inferior, nasal, and inferotemporal parafoveal regions most commonly affected, while the superior quadrants often showed no significant changes in the early stages [12,23,25,27]. This sectoral distribution was further supported by the OCT-derived indices demonstrating a higher diagnostic performance in the inferior ETDRS sectors [31].

Several studies reported an association between OR thinning and HCQ exposure metrics, particularly, cumulative dose or long-term treatment. Significant or dose-dependent relationships were described in multiple cases [9,10,11,17,18]. Longitudinal data further suggest that retinal thickness may remain stable for extended periods before accelerated thinning occurs beyond a critical exposure threshold [10].

Advanced imaging studies provided additional structural detail. Garg et al. reported the attenuation of PR outer segments using visible-light OCT, with the cone outer segments affected earlier than rods [13]. Cakir el al. observed differences in the PR segment length in the nasal and temporal regions related to the treatment duration, supporting early PR structural involvement [26].

Despite these convergent observations, not all studies demonstrated a clear correlation between OR changes and the treatment duration or cumulative dose. Trenkic Bozinovic et al. found no significant correlation with the HCQ duration, although greater inner and outer foveal thinning was observed in eyes with established retinopathy compared with those without visible macular changes [25]. Similarly, Allahdina et al. reported no significant differences in treatment duration, cumulative dose, or daily dose between patients with and without retinopathy, despite identifying an increased ONL reflectivity and elevated OCT-based metrics in the affected eyes [31].

Variability was also noted regarding which OR parameter was the most sensitive, with some studies emphasizing ONL thinning [12,20,23], others reporting reduced ORT [9,15], and others focusing on PR segment alterations [13,21,26]. These differences likely reflect the heterogeneity in the imaging protocols, segmentation approaches, and disease stage rather than contradictory biological effects.

Therefore, parafoveal ONL thinning and sectoral OR attenuation are supported as the most consistent structural OCT findings associated with HCQ exposure. The discrepancies across studies mainly concern the strength of the dose correlations and the specific OR metrics used, rather than the presence of early OR involvement itself. These findings support the role of OR OCT parameters as sensitive biomarkers for the early detection and longitudinal monitoring of HCQ-related retinal toxicity.

### 3.2. Elipsoid Zone

Accumulating evidence suggests a sequential pattern of OR involvement in HCQ retinopathy, in which the disruption of the EZ represents the earliest and most sensitive structural correlate of functional impairment, followed by progressive alterations in the external limiting membrane (ELM) and, ultimately, the RPE, the latter serving as a marker of advanced and often irreversible disease. Once the EZ integrity is compromised, the eyes demonstrate continued progression despite drug cessation, with a subsequent ELM disruption indicating a more severe OR compromise and poorer prognosis. Table 2 summarizes the published studies that analyze the EZ integrity.

Multiple studies reported that early HCQ retinopathy exhibits a preferential parafoveal or pericentral EZ involvement, with a progression toward RPE damage in more advanced stages. He et al. observed a pericentral pattern in all early cases, with initial EZ damage followed by RPE involvement, and associated visual acuity reduction [36]. Similarly, Kim et al. reported that pericentral involvement was the most frequent pattern, followed by a parafoveal and mixed distributions, with the inferior temporal sectors most commonly affected [40].

Several investigations identified EZ disruption as a marker of disease severity and progression. Jayakar et al. emphasized that EZ loss reflects significant photoreceptor loss and severe toxicity, while Ahn et al. demonstrated that an increasing EZ defect length and reduced fovea PR defect distance were reliable indicators of progression [42,44]. Quantitative thresholds were also proposed, with one study reporting that a partial EZ attenuation of ≥1.9% achieved a high sensitivity and specificity for toxicity detection [41].

EZ changes were repeatedly shown to correlate with functional impairment, including reduced visual acuity and visual field sensitivity [36,42,44]. Structural–functional correspondence was further supported by studies reporting concordant OCT and visual field abnormalities in parafoveal configurations [42,43]. Kim et al. used a clock-hour analysis of parafoveal OCT scans to map the distribution of HCQ-related OR changes. They found that EZ and RPE alterations were not uniform but demonstrated preferential involvement in certain clock-hour sectors, particularly in the inferotemporal and superotemporal parafovea, regions known to correspond with early functional deficits on VF testing. This topographic approach provides additional sensitivity in detecting localized early toxicity and underscores the value of a spatially resolved analysis when monitoring patients at risk [40].

Despite the usefulness of the EZ in detecting HCQ retinopathy, it requires a subjective evaluation of the damage extent, being, therefore, subjective to errors such as observer-related errors. Talcott et al. introduced the EZ At-Risk parameter as an automated OCT biomarker for HCQ retinopathy, quantifying areas of subclinical EZ alteration likely to undergo progression. In a cohort of HCQ users, the mean EZ At-Risk burden was significantly greater in eyes with established toxicity compared with non-toxic HCQ eyes and healthy controls, and this parameter demonstrated a strong association with the HCQ dosage when normalized to both the actual and ideal body weight. These findings support the utility of automated EZ metrics for early risk stratification and objective monitoring. The authors suggest that additional research is required to clarify the longitudinal behavior of this novel biomarker in patients receiving HCQ, to compare it with other quantitative measures, to investigate potential threshold values relevant for toxicity risk stratification, and to strengthen correlations with disease severity [38].

In a similar manner, Ugwuegbu et al. applied a semi-automated segmentation approach to OCT macular cube scans, deriving volumetric and en-face measurements of ORL. Eyes with HCQ retinopathy demonstrated a significant thinning of the ONL/HFL-EZ complex in parafoveal regions as well as quantifiable EZ-RPE attenuation on en-face mapping, changes that were detectable even in early disease. A longitudinal assessment further confirmed the progressive structural decline in the affected eyes [48].

These studies highlight the potential of both automated and semi-automated OCT-derived biomarkers to identify the early structural alterations, quantify the disease burden, and monitor the progression beyond conventional clinical and functional testing.

Yucel Gencoglu et al. reported a reduced reflectivity of the EZ, ELM, and RPE in the parafoveal and perifoveal regions of HCQ users compared with controls, while the central foveal reflectivity was often preserved or even increased. Reflectivity losses in the ELM and RPE were most pronounced in eyes with an established EZ disruption, and such alterations were closely associated with worse visual outcomes, highlighting their prognostic significance [37].

Evidence across multiple studies supports EZ disruption as a pivotal diagnostic marker in HCQ retinopathy, representing a critical transition from sub-clinical structural alteration to clinically relevant disease. Partial attenuation or focal EZ discontinuity consistently precedes ELM and RPE involvement, and shows strong correlations with VF sensitivity loss and reduced VA. Importantly, EZ integrity appears to predict disease progression even after drug discontinuation. While the traditional EZ assessment relies on qualitative interpretation, emerging automated and quantitative metrics offer new directions for objective risk stratification and longitudinal monitoring.

### 3.3. Inner Retinal Alterations

Across studies, findings related to the inner retinal layer (IRL) involvement in HCQ exposure are more heterogeneous than those reported for OR alterations. While several studies identified the thinning of the GCL, GCIPL, or RNFL, others failed to detect significant IR changes, particularly in early or asymptomatic patients (Table 3).

Multiple investigations reported GCL or GCIPL thinning in HCQ-treated patients, including those without clinically evident retinopathy, suggesting that IR involvement may occur before overt toxicity in the selected cohorts [51,53,63,65]. Sectoral thinning was frequently observed, affecting the parafoveal or perifoveal regions, rather than the foveal center [53,60]. Sonalcan et al. reported a reduced mean and temporal RNFL thickness as well as decreased GCIPL thickness in HCQ users, although no differences were observed between HCQ subgroups stratified by treatment duration [51].

Several studies found associations between IR thinning and a longer HCQ exposure or higher cumulative dose. Agcayazi et al. reported correlations between GCL thinning and HCQ intake duration, while Kim et al. observed macular GCL and RNFL thinning predominantly in patients with established retinopathy [53,54]. Godinho et al. found a negative correlation between HCQ treatment duration and foveal GCL thickness, further supporting a potential exposure-related effect [58].

Longitudinal and severity-based analyses suggest that IR thinning may be more prominent in advanced disease stages. Membreno et al. showed that IR thinning was largely absent in early disease but became evident in more severe HCR groups, whereas OR thinning was present even in mild cases [56]. Similarly, Mondal et al. reported damage involving both IR neurons and ORL in long-term HCQ users [55].

In contrast, several studies reported no significant IR differences between HCQ-treated patients and controls. Jung et al. found no differences in macular GCL thickness between groups despite detecting early functional abnormalities on mfERG [50]. Mimier Janczak et al., similarly, reported no OCT detectable differences in retinal, RNFL, GCL, or GCIPL thickness between SLE patients receiving HCQ and controls, and no correlation between HCQ treatment duration and retinal parameters [52].

Findings vary when analyzing IR involvement in HCQ retinopathy. While some studies did not report any significant findings regarding GCL thickness between HCQ patients and controls [23,50,52], others found an early thinning in the perifoveal GCL thickness as a sign of early degeneration [53,65], with signs of correlations between HCQ intake and IR thinning [57,58,60]. IR thinning appears more consistently associated with advanced or established toxicity, suggesting that IR OCT parameters may function as markers of disease severity rather than early detection. Peripapillary retinal nerve fiber layer measurements show limited diagnostic reliability and should not be used in isolation for toxicity screening [62,63,64].

### 3.4. Choroid

Studies showed that, in HCQ retinopathy, the first affected layer in the choroid is the choriocapillaris (CC), objectifying the importance of OCT examination of this area (Table 4). The CC is the source of nutrients for the ORL, including RPE and PRL.

Choroidal thickness (CT) findings in HCQ-intake users are heterogeneous, with reports of both choroidal thinning and relative thickening depending on cohort characteristics, disease activity, and retinopathy status. Overall, the data suggest that choroidal impairment is more consistently observed in established or advanced HCQ retinopathy, whereas earlier-stage or short-duration HCQ cohorts show more variable patterns. While most authors declared a lower CT to be associated with HCQ intake, the underlying disease may lead to other choroidal modifications that could either mask or influence these results. Braga et al. found an increase in CT in SLE associated with lupus nephritis that was not linked to disease duration [75].

Several studies reported reduced CT or signs of choroidal impairment in eyes with HCQ retinopathy, particularly in advanced disease. Ahn et al. found significantly thinner CT at most measured locations in eyes with HCQ retinopathy compared with eyes without, with severity significantly associated with reduced CT. CT was also lower in areas exhibiting OR defects compared with areas without [73]. Halouani et al. similarly reported lower subfoveal and mean choroidal parameters in advanced retinopathy compared with controls, supporting the concept of choroidal involvement in more severe disease [68].

Additional studies suggested a link between HCQ exposure and reduced choroidal metrics. Hasan et al. reported a relationship between an increased cumulative HCQ dose and decreased choroidal volume, and found that choroidal vascular parameters were significantly lower in the study group than controls [67]. Polat et al. reported reduced subfoveal CT in HCQ patients compared with controls, and further described decreased temporal CT in patients with >5 years of HCQ use. These results suggest that early parafoveal RPE thickening may represent an adaptive or preclinical structural response to drug exposure, preceding the reflectivity loss and disruption observed in manifest toxicity [71].

Arias-Peso et al. reported variable CT alterations in patients with SLE under HCQ treatment. Their results indicate that patients treated with HCQ for less than five years exhibited a thicker choroid compared to control subjects, particularly in the central, nasal, and superior sectors. Conversely, individuals receiving HCQ therapy for more than five years demonstrated a significant reduction in CT, suggesting a thinning effect associated with prolonged treatment duration. Their results also revealed sectoral differences, with thinner CT observed in the temporal and inferior regions in association with HCQ exposure. Moreover, a positive correlation was identified between disease activity (measured by the SLEDAI score) and HCQ dosage, implying that higher doses of HCQ may be required to achieve adequate disease activity control [66].

The relationship between CT and HCQ exposure metrics remains inconsistent. Ahn et al. reported a positive correlation between subfoveal choriocapillaris thickness and HCQ duration and cumulative dose adjusted for body weight [73], while Ru reported no correlation between CT and HCQ duration or cumulative dose in the pediatric population [69]. One study reported a decreased choroidal volume with a higher cumulative dose [67], while another emphasized the influence of disease activity and short-duration thickening patterns [66].

Choroidal involvement in HCQ appears to reflect drug-related effects with disease-specific inflammatory or microvascular processes. While several studies report reduced CT, CVI, and CC perfusion in patients with long-term HCQ exposure or established retinopathy, others describe early CT potentially related to inflammatory activity. These discrepancies highlight the susceptibility of choroidal metrics to systemic confounding factors. Consequently, OCT parameters may serve as adjunctive biomarkers of disease progression rather than primary diagnostic indicators when analyzing the choroid, with CVI emerging as a potentially more stable metric than absolute thickness measurements.

### 3.5. OCT-A Findings

The evaluation of retinal vascular alterations using optical coherence tomography angiography (OCT-A) has become increasingly important in the early detection of HCQ-induced retinal toxicity. OCT-A provides a non-invasive, high-resolution assessment of the retinal microvasculature, allowing a detailed visualization of both superficial (SVP) and deep (DVP) capillary plexuses. Since vascular affliction may precede overt structural or functional changes detectable using imaging or VF testing, OCT-A can serve as a sensitive biomarker for subclinical toxicity. A quantitative analysis of the vessel density (VD) and perfusion parameters enables the identification of subtle microvascular disturbances, particularly in the parafoveal and perifoveal regions, which are preferentially affected in HCQ toxicity. Table 5 offers the parameters of the published studies that analyzed OCT-A in HCQ patients.

Most studies agree on a decrease in VD across the SVP and DVP in patients with HCQ intake > 5 years. While some authors found a decrease in VD compared to healthy controls regardless of treatment duration [93], other studies support these findings by objectifying a correlation with the cumulative HCQ dosage regardless of treatment duration [90]. However, there are separate studies that suggest the lack of any type of vascular involvement in HCQ toxicity, with their studies finding no statistical difference between the HCQ and control groups [83], or even an increase in VD in the HCQ groups compared to controls.

Leclaire et al. highlighted that SLE patients are predisposed to a reduction in retinal VD due to pathological alterations caused by the disease and not HCQ toxicity [79]. Subasi et al. investigated the retinal microvascular changes in patients with SLE, reporting significant reductions in VD within both SVP and DVP compared to healthy controls. The decrease was particularly pronounced in the perifoveal regions of the deep plexus, while the superior parafoveal and temporal areas were relatively spared. Furthermore, a subgroup analysis revealed no structural differences between SLE patients with or without renal involvement, though perifoveal DVP alterations in the superior quadrant were more evident in those with nephropathy, indicating potential diagnostic value for systemic disease activity [86]. These results suggest that microvascular impairment in SLE primarily affects the deep retinal circulation and may reflect disease-related vascular pathology rather than HCQ toxicity, underscoring the relevance of OCT-A in the early detection of subclinical vascular changes in SLE.

Yu et al. reported a reduction in retinal microvascular density in SJS patients, with and without HCQ treatment, compared to healthy controls, with an even greater decrease observed in the HCQ-treated group. Visual acuity was significantly reduced in both the SJS and HCQ groups relative to controls, and more severely affected in patients receiving HCQ. A quantitative OCT-A analysis further revealed reductions in both the superficial and deep microvascular indices in the HCQ group compared to SJS alone. VD was significantly decreased in multiple parafoveal and perifoveal sectors and in the central regions in SJS versus controls, with more extensive reductions in the HCQ-treated cohort [85]. These findings highlight OCT-A-derived VD as a promising non-invasive biomarker for evaluating disease severity and vascular compromise in SJS and its modulation by systemic therapy.

Similarly, Liu et al. observed comparable microvascular alterations in patients with RA, with further reductions associated with long-term CQ therapy. Their study demonstrated that RA patients exhibited significantly decreased retinal VD compared to healthy controls, particularly within the SVP, while those treated with CQ showed an additional decline in both SVP and DVP parameters. The reduction in VD was most evident in the parafoveal and perifoveal regions, as well as in the superior and temporal retinal sectors. Conjunctival VD was likewise diminished in both RA- and CQ-treated groups, suggesting that the microvascular compromise extends beyond the retina. These findings indicate that CQ exposure may exacerbate the retinal and conjunctival vascular rarefaction in autoimmune disease, and further support the utility of OCT-A as a sensitive imaging tool for detecting early, subclinical vascular changes related to disease progression and antimalarial drug toxicity [84].

Multiple studies observed an increase in the foveal avascular zone (FAZ) size which was positively correlated with the cumulative HCQ dose and duration. Furthermore, a decrease in FAZ VD in both SVP and DVP has been objectified in patients with >5 years of HCQ intake. Despite these findings, alterations in FAZ did not correlate with clinical symptoms as there were no significant differences when evaluating VD parameters between normal and abnormal mfERG in HCQ patients, suggesting that microvascular alterations could appear as a marker of subclinical toxicity or as a consequence of disease activity [89].

The use of inter-eye symmetry may provide additional data regarding VD as macular VD was shown to be reduced in HCQ patients compared with healthy controls in the absence of HCQ retinopathy [76].

Halouani et al. investigated CC alterations in patients receiving long-term HCQ therapy and found significant flow-related deficits (FDs) in eyes with HCQ retinopathy. Compared with both HCQ-treated eyes without signs of toxicity and healthy controls, eyes with confirmed HCQ retinopathy showed a significantly higher percentage of CC FD, a larger mean FD size, and a lower FD number, indicating more extensive and confluent areas of CC nonperfusion. The total area of flow deficits was also increased in the toxicity group relative to controls, whereas no significant differences were observed between the non-toxicity and control groups for most CC parameters. These findings provide strong evidence of CC involvement in the pathophysiology of HCQ-induced retinal toxicity, suggesting that CC flow impairment may occur secondary to OR and RPE damage or as a direct vascular effect of the drug. The authors proposed that a quantitative CC flow-deficit analysis may offer valuable insight into the microvascular alterations underlying HCQ retinopathy and may aid in detecting early choroidal involvement before irreversible structural damage develops [87].

The OCT-A studies highlight microvascular alterations in HCQ patients, particularly the reduced VD and enlarged FAZ after prolonged exposure. However, the interpretation of these findings is complicated by the intrinsic microvascular involvement of underlying autoimmune diseases, such as SLE and RA. While the reduced macular VD and CC hypoperfusion may reflect an early-toxicity-related vascular compromise, these changes lack sufficient specificity when considered in isolation. Therefore, OCT-A parameters should be interpreted as complementary diagnostic biomarkers, integrated with structural OCT findings and clinical context, rather than used as standalone screening tools.

## 4. Discussion

HCQ retinopathy represents a diagnostic challenge because structural retinal damage may develop insidiously and progress despite treatment discontinuation, often before patients report visual symptoms. The present narrative review synthesizes the current evidence on OCT- and OCT-A-derived biomarkers associated with HCQ exposure, highlighting their respective diagnostic roles, limitations, and clinical applicability across disease stages.

When considered together, the available evidence indicates that structural OCT demonstrates a greater consistency and robustness than OCT angiography (OCT-A) for the detection of HCQ-related retinal toxicity, particularly in the early or subclinical stages. Across multiple studies, OCT-derived structural biomarkers, most notably, sectoral OR, ONL thinning, and EZ alterations, show reproducible patterns that correlate with cumulative exposure, disease stage, and functional impairment. In contrast, the OCT-A findings, including reduced macular VD and FAZ enlargement, exhibit substantial inter-study variability and are strongly influenced by the underlying autoimmune disease, inflammatory activity, and imaging methodology.

While OCT-A can reveal microvascular alterations and may detect changes in selected high-risk or advanced cases, these vascular metrics lack specificity for HCQ toxicity when used in isolation. Consequently, OCT-A appears best suited as a complementary modality, providing supportive microvascular information alongside structural OCT, rather than as a stand-alone screening tool. This hierarchical performance supports current screening strategies that prioritize structural OCT while reserving OCT-A for selected cases requiring additional pathophysiological or prognostic insight.

Membreno et al. proposed a classification of HCQ retinopathy severity based on the extent of EZ disruption measured on OCT. Patients were divided into four groups: group 1, with <100 μm of EZ loss; group 2, with 100–1000 μm of EZ loss; group 3, with >1000 μm of EZ loss but the preservation of a central foveal EZ island > 500 μm; and group 4, with <500 μm of residual foveal EZ. Their analysis showed that groups 1 and 2 did not differ significantly from the control subjects in terms of visual acuity, whereas patients in groups 3 and 4 exhibited measurable declines in visual function [56]. In a similar approach, Allahdina et al. categorized the affected eyes into four OCT-based stages: stage 1 with subtle parafoveal alterations, stage 2 with definite localized parafoveal damage but preserved fovea, stage 3 with extensive parafoveal involvement and intact fovea, and stage 4 with foveal involvement. A longitudinal follow-up after drug cessation revealed that eyes in stages 1 and 2 generally maintained stable VA and VF sensitivity, with stage 1 cases even demonstrating functional improvement on mfERG parameters. By contrast, eyes in stages 3 and 4 showed progressive structural deterioration and significant vision loss. Notably, in stages 2 and 3, the length of the central EZ island progressively shortened in the majority of eyes (approximately two-thirds of stage 2 and nearly nine-tenths of stage 3), indicating that, beyond the earliest stage, the discontinuation of HCQ does not fully prevent retinopathy progression [28]. This aspect aligns with other studies that confirm that HCQ maculopathy advances despite drug cessation [25,29,44].

The 2016 screening recommendations for CQ and HCQ retinopathy proposed by the Marmor et al. support screening at baseline followed by annual screening after 5 years for patients without additional risk factors such as a high daily dosage (>5 mg/kg real body weight for HCQ and >2.3 mg/kg real body weight in CQ), a longer duration of use (>5 years), concomitant renal disease with a subnormal filtration rate, the use of other retinotoxic drugs such as Tamoxifen, or the presence of a concomitant macular disease that could influence susceptibility to HCQ retinopathy. In these high-risk patients, screening should be performed annually [98]. The recently published 2025 revisions continue to follow these recommendations, adding initiation at an advanced age as another high-risk factor. They propose annual screening using OCT, wide-pattern autofluorescence as the primary annual screening techniques with visual field and ERG as secondary, confirmatory techniques, while OCT-A was not recommended for annual screening [99].

Some societies recommend that the initial ophthalmological consult not be mandatory for HCQ toxicity screening, as the clinical benefit is insufficient to justify the required costs [100], while others still recommend baseline testing to rule out confounding diseases [101]. These contradictory statements arise from the socio-economic costs that may burden the national health services, therefore highlighting the need for better toxicity biomarkers. Furthermore, a lack of baseline OCT data useful in future comparisons may lead to a false-positive HCQ retinopathy diagnosis, taking the patient off an effective, well-tolerated treatment regimen and starting more expensive treatments which require additional investigations [102].

Despite the existing protocols and guidelines, multiple atypical presentations have been reported, spanning from rapid onset [103,104,105,106,107], peripheral alterations [108,109], and unilateral or asymmetric ocular toxicity [110,111]. While the recommendations for screening are after 5 years of HCQ therapy, other confounding factors may lead to faster toxic retinal modifications [112,113]. A retrospective analysis performed on 95 Asian patients diagnosed with HCQ retinopathy over 13 years identified 14 cases with atypical presentations, with the authors highlighting the need for vigilance when performing screening for HCQ toxicity [114].

Mohapatra et al. identified nine cases of accelerated HCQ toxicity, the fastest appearing in 2 months of drug intake at a cumulative dose of 18 g, with the other eight cases appearing in under 12 months of treatment [107]. Similar reports [103,104,105,106] have been published with early macular toxicity that may have been caused by an increased bioavailability of HCQ due to decreased cytochrome p450 enzyme activity that could be affected by other medications such as nonsteroidal anti-inflammatory drugs or genetic predisposition. Lee et al. highlighted the existence of genetic polymorphisms in Korean SLE patients taking HCQ that led to different HCQ blood level concentrations despite a similar prescribed dosage [115].

While Petri et al. found a relationship between HCQ blood levels and toxicity [116], other studies analyzed HCQ blood levels to correlate with retinal structural and vascular toxicity but did not observe any significant relationships between these parameters [80,81,92]. Liu et al. highlighted that SLE patients with a high concentration of blood HCQ presented a better controlled disease activity, being negatively correlated with disease severity scores and inflammation markers [81].

Multiple studies that focused on the incidence of HCQ retinopathy among different populations reported that the prevalence of maculopathy in recent years was lower than previously reported [39,46,117]. Different Asian populations reported a pericentral or mixed pattern in the progression of HCQ retinopathy [33,35,36,40,44,73,118], drawing attention to the need for a wider structural and functional analysis in these populations compared to the standard “bull’s eye” maculopathy [34]. Short obese women may be more susceptible to HCQ overdose as an ideal body weight may be better in evaluating the daily dose in these patients. Daily dosing based on the older 6.5 mg/kg ideal weight threshold is safer in women with a BMI of 30 kg/m^2^ or more [47,119].

In contrast to the initial thickening of OR structures seen in patients with drusen-like deposits (DLDs), subsequent degenerative processes may lead to the thinning of the ONL, photoreceptor layer (PRL), and other ORL. As the inflammatory insult from vasculitis subsides or becomes chronic, ischemia, immunocomplex deposition, and microvascular compromise may trigger the apoptosis or shrinkage of PR cell bodies and supporting Müller cells. Over time, this secondary degeneration leads to measurable reductions in ONL thickness and PRL thickness. In parallel, decreased perfusion and chronic stress may impair the RPE and outer segment integrity, further contributing to the structural atrophy of the OR. These thinning changes may lag behind the earlier thickening, but mark a transition from active inflammation toward permanent tissue loss. Although Kitay et al. observed no significant change in mfERG between groups, implying a preserved cone function despite structural differences, the structural thinning could signal the declining resilience of PR before the functional loss becomes detectable [14].

Kukan et al. found that SLE patients with DLD exhibited lower CVI values compared to healthy controls, while no significant difference was observed between SLE eyes with and without DLD. Eyes with DLD showed a thicker GCL but the thinning of the OR and PR relative to those without DLD, indicating concomitant neurodegenerative changes. The authors proposed that the presence of DLD may reflect a more active inflammatory disease state, characterized by choroidal vascular alterations associated with immune complex deposition and vasculitis-type processes. These findings suggest that DLD could serve as an indicator of heightened inflammatory activity and evolving retinal degeneration in SLE [70].

Zirtiloglu et al. evaluated the RNFL thickness and radial peripapillary capillary (RPC) VD in patients with systemic sclerosis (SS) using OCT-A and found no significant overall differences compared with healthy controls. However, several microstructural and vascular parameters showed correlations with disease activity and treatment profiles. Specifically, the superior RNFL, whole, peripapillary, superior and inferior RPCvalues significantly decreased with increasing antinuclear antibody titers, while the nasal RNFL thickness declined with a longer disease duration, suggesting a progressive microvascular compromise with the disease severity. Interestingly, patients using HCQ demonstrated higher RPC inside values than those not on HCQ, and the RPC whole, RPC peripapillary, and RPC nasal densities were significantly higher in corticosteroid users compared to non-users [120]. These results indicate that, although global peripapillary perfusion and RNFL thickness remain preserved in SS, localized vascular attenuation correlates with serologic disease activity and duration, whereas certain systemic therapies such as HCQ and corticosteroids may exert a protective or modulatory effect on peripapillary microcirculation.

HCQ retinopathy has been shown to have racial variations. While, most often, the defect follows a parafoveal pattern, being a specific clinical finding, Melles and Marmor found that, in the Asian population, it can have a pericentral disposition [121]. This finding highlights the need for an extensive level of attention when evaluating HCQ toxicity in certain populations as smaller OCT B-scans or 10-2 perimetry may overlook existing peripheral lesions and delay drug cessation.

Understanding the mechanisms of HCQ toxicity may provide additional guidelines that could be used in the pediatric population. Bousquet et al. reported a case of toxic retinopathy in a 23-year-old patient undergoing under 200 mg/day HCQ for 13 years, finding specific retinal damage on OCT, highlighting the need for screening guidelines in the pediatric population [122]. Lu et al. draw attention to the need for a close ophthalmological examination in order to diagnose adverse effects in children, as, in spite of the low HCQ dose, renal impairment caused visual loss in a 14-year-old patient [123]. A group led by AlAhmed evaluated the screening rate for HCQ retinopathy in the pediatric rheumatology department and developed an algorithm that led to the increase from 65% to 85% in 12 months, emphasizing the importance of interdisciplinarity in increasing awareness among the medical provider and patients [124].

Ru et al. found that CT is lower in juvenile SLE under HCQ than in heathy age-matched controls with a negative correlation between CT and the systemic cytokine profile [69]. Given that alterations in CT have been shown to appear secondary either to HCQ or to the autoimmune disease itself, in the absence of structural and/or functional retinal damage, CT cannot be used alone as a biomarker. Furthermore, physiological fluctuations have been shown to influence CT [125]; therefore, it has a low reliability in detecting HCQ toxicity, especially in the presence of a systemic inflammatory disorder.

Cystoid macular edema has also been reported in rare cases as a side effect of HCQ treatment. A proposed mechanism is that the accumulation of HCQ in the RPE provokes a disruption in the pump function that will lead to intraretinal fluid accumulation [126,127]. Given this mechanism, carbonic anhydrase inhibitors have been used to increase the RPE pump function and stimulate fluid absorption [128]. Dexamethasone has also been suggested in refractory cases [32,129].

While HCQ is mainly used in autoimmune and inflammatory diseases, it has been described in other pathologies as well. The SARS-CoV-2 pandemic caused an increase in the search of new immunomodulatory medicine, HCQ being proposed as a prophylactic drug used to reduce the risk of severe acute respiratory syndrome [130]. Clinical trials have described using HCQ as an adjunctive therapy for advanced metastatic melanoma in association with other drugs, now known to cause retinal toxicity [131]. Understanding the molecular mechanisms and evolution of HCQ retinopathy may also lead to safety profiles regarding the use of this drug in other diseases.

### 4.1. Toward a Diagnostic Hierarchy and Personalized Screening

The collective evidence supports a hierarchical diagnostic framework in which structural OCT biomarkers, particularly the ORT metrics and EZ integrity, form the foundation of early toxicity detection. IR and choroidal changes provide additional information regarding disease severity and progression, while the OCT-A findings offer complementary insights into vascular involvement. This layered approach aligns with current screening recommendations and underscores the importance of integrating multiple imaging domains rather than relying on single parameters.

Advances in automated OCT analysis and artificial-intelligence-assisted segmentation hold promise for improving reproducibility, reducing observer variability, and enabling personalized screening strategies.

Future automated machine-learning techniques are required for the proper quantification of EZ loss, RPE damage, and the effects of the systemic autoimmune disease on the retina. While some studies have described using automated machine learning for evaluating HCQ toxicity [38,41,43], researchers have attempted to use automated retinal vessel analysis to measuring venular dilation and assess rheumatic disease inflammatory activity [132]. Quantitative metrics such as automated EZ attenuation, ORT mapping, and standardized sectoral analysis may facilitate earlier detection and more accurate risk stratification in patients receiving long-term HCQ therapy.

### 4.2. Limitations

This review has several limitations. As a narrative synthesis, it does not provide quantitative pooled estimates or a formal risk-of-bias assessment. Considerable heterogeneity exists among the included studies with respect to the imaging protocols, segmentation algorithms, patient populations, and definitions of toxicity. Additionally, the lack of a separate-disease-grouped analysis may lead to confounding effects of the underlying autoimmune disease activity that limit the specificity of our findings. Nevertheless, the consistency of key structural OCT biomarkers across diverse cohorts strengthens the clinical relevance of the conclusions drawn.

Most included studies were observational in design, predominantly cross-sectional or retrospective, which limits causal inference and the ability to assess the temporal progression or predictive performance of individual biomarkers. Variability in OCT and OCT-A devices and technology, image acquisition parameters, and post-processing methods further complicates the direct comparison of outcomes and may contribute to inconsistent findings, particularly for the choroidal and microvascular parameters. Moreover, OCT-A-derived vascular metrics are especially susceptible to disease-related and systemic confounders, underscoring the need for cautious interpretation when attributing these changes solely to HCQ toxicity.

Future prospective studies using standardized imaging protocols and unified diagnostic criteria are required to validate proposed biomarkers and to clarify their role in risk stratification and screening strategies.

## 5. Conclusions

HCQ retinopathy is a potentially vision-threatening complication that is correlated with the cumulative dose, treatment duration and specific risk factors. This narrative review highlights the role of OCT-derived structural biomarkers in the early detection and monitoring of HCQ-related retinal toxicity.

Alterations in the ORL, particularly EZ attenuation and ONL thinning, could be used as indicators of early toxicity, often preceding functional impairment. IR and choroidal changes appear to reflect more advanced disease stages and provide complementary information regarding severity and progression. OCT-A VD metrics have been considered useful in revealing microvascular alterations associated with prolonged exposure; however, these findings lack sufficient specificity when considered in isolation and should be interpreted within the context of structural OCT findings and the underlying autoimmune disease activity. An integrated, multimodal imaging approach that prioritizes structural OCT biomarkers and incorporates OCT-A as a complementary tool offers the most reliable diagnostic strategy for HCQ retinopathy.

While current OCT-derived biomarkers show promise in detecting HCQ retinopathy, the future implementation of automated OCT analysis and artificial-intelligence-assisted biomarker quantification may enhance screening precision, reduce observer variability, and enable personalized risk stratification, ultimately improving patient safety and long-term visual outcomes.

## Figures and Tables

**Table 1:** Published studies that analyze HCQ toxicity using OCT focused on OR alterations.

Author	Participants	Medication Duration (Years)	Cumulative HCQ Dose (g)	Mean Daily Dose to Real Body Weight (mg/kg)	Key Findings
Farvardin, 2024 [11]	47 HCQ patients, 25 controls	5.1 ± 5.2	301 ± 365	/	Link between ORL thickness and HCQ cumulative dose. Evaluating MT in patients without OCT toxicity signs is not useful in detecting early HCQ toxicity.
Salameh, 2024 [12]	40 HCQ eyes vs. 40 controls	8.56 ± 4.4	1215.45 ± 654.77	/	ONL thinning in parafoveal and perifoveal regions on OCT. Most pronounced in inferior quadrants of inner and outer zones. No significant changes in central or superior quadrants. Weak correlation between nasal inner ONL thickness and cumulative HCQ dose. Foveal thinning observed over time between initial and latest OCT.
Montesel, 2024 [9]	35 HCQ eyes, 30 controls	8 ± 5.3	704.3 ± 522.5	/	Thinner ORL layers in HCQ patients indicate early structural changes. Dose-dependent associations support OCT parameters as reliable early biomarkers. Early OCT detection may prevent progression to foveal involvement. Important due to severity-dependent progression of HCR.
Garg, 2024 [13]	10 HCQ patients and 12 controls	14.3 ± 4.1	1723.6 ± 574.2	/	Earlier diagnosis of HCQ toxicity can be made by using VIS-OCT imaging. Attenuation of the putative cone OS, followed by the putative rod OS, suggests that cone PR are preferentially affected over rods.
Kitay, 2023 [14]	G1: 34 HCQ eyes with DLDS and G2: 32 HCQ eyes without DLDs	G1: 8.20 ± 5.44 G2: 6.10 ± 4.89	G1: 608.60 ± 361.48, G2: 646.16 ± 491.62	/	DLDs affect MT structurally but not PR functional transmission. Foveal ORT significantly increased in SLE + DLD vs. SLE without DLD. PRL significantly thinner in DLD patients. PRL thinning likely due to degeneration after inflammation, vasculitis, and reduced blood flow.
Melles, 2022 [10]	301 HCQ patients G1: 219 with stable thickness G2: 82 with rapid thinning	G1: 14.7 ± 5.4 G2: 15.4 ± 5.4	/	G1: 3.4 ± 1.1 G2: 4.2 ± 1.2	RT remains stable for many years in most patients receiving long-term HCQ therapy, but, after a critical point, the retina may begin to thin rapidly. Sequential plots of inner and outer ETDRS ring MT provide objective evidence of this early structural change several years before conventional signs appear.
Tarassoly, 2022 [15]	53 HCQ eyes, 21 controls	11 ± 4	1114 ± 481	/	Mean ORT lower in HCQ group vs. controls. No difference in IRT between groups. In 32% of eyes, FT thinning is explained by thin IR.
Borrelli, 2021 [16]	G1: 63 HCQ eyes G1a: 46 eyes patients without HCR G1b: 17 HCR G2: 30 controls	G1: 8.4 G1a: 7.6 G1b: 10.5	G1: 782.8 G1a: 704.3 G1b: 973.4	/	HCQ patients show significant thinning of inner and outer retina. Eyes without clinical retinopathy exhibit INL and ONL thinning, indicating early retinal cell loss. Retinal thinning correlates with macular dysfunction on mfERG in HCQ patients without retinopathy. Structural changes from HCQ toxicity may precede clinically detectable retinopathy.
Sallam, 2021 [17]	G1: 60 HCQ eyes G1a: 10 with normal VA G1b: 50 with decreased VA G2: 50 controls	G1a: 5.6 G1b: 8.3	G1a: 408.8 G1b: 1214.7	/	OCT parameters are more affected in the higher-HCQ-dose group. OCT alterations related to long-term HCQ exposure occur before clinically detectable retinopathy and may be associated with a mild reduction in VA. VA was primarily influenced by daily HCQ dose, duration of exposure, FT, and disruption of the PRL.
Manoj, 2021 [18]	110 patients	7.7 ± 2.5	840 ± 305.6	14.8 ± 5.4	Retinopathy was associated with color vision defects and parafoveal/perifoveal thinning. Significant correlation between high daily HCQ dose and retinopathy presence.
Kim 2021 [19]	1723 HCQ eyes	15.9 ± 6.6	1436.7 ± 529	5.15 ± 1.53	The RT deviation map demonstrated excellent sensitivity and specificity for the detection of HCR.
Hasan, 2020 [20]	100 HCQ patients > 5 y HCQ, 70 controls	6.3	/	/	“Flying saucer fovea” on OCT is an early indicator of HCR. Severe cases show PR inner–outer segment loss and RPE atrophy. ONL volume strongly predicts VF-RT loss. HCQ toxicity affected the superior parafoveal, superior peripheral, inferior parafoveal, and nasal parafoveal areas of the fovea.
Pandey, 2020 [21]	G1: 167 HCQ patients G1a: 9 HCR patients	G1a: 4.5 ± 2.96	G1a: 515.1 ± 360.7	G1a: 6.09 ± 1.79	HCQ associated with decreased OS and IS thickness, and GCIPL thinning. Increased RPE thickness observed. IS–OS disruption, PRL disruption, ID zone loss, and RPE loss was observed in HCR. Hyperreflective choroid seen secondary to RPE loss.
Dias-Santos, 2020 [22]	G1a: 18 HCR patients G1b: 143 without HCR	G1a: 14.7 G1b: 6.9	G1a: 2130.1 G1b: 939	/	HCR patients presented PR thinning with corresponding VF defects. Other findings included RPE detachment and cystoid macular degeneration.
Casado, 2020 [23]	42 HCR eyes, 72 controls	3.44	/	/	HCR patients show decreased ONL thickness in nasal macula and inferior sectors.
Souza Cabral, 2019 [24]	G1: 217 patients with antimalaria treatment G1a: 9 HCR patients	G1a: 10.4	/	/	Only patients with advanced stage maculopathy presented abnormalities during the ophthalmologic exam. HCR was objectified on OCT as different degrees of alteration at the junction of the internal and external segments of the retina and RPE.
Trenkic Bozinovic, 2019 [25]	G1: 26 HCQ eyes with no visible changes in the macula G2: 30 HCR eyes	6.11 ± 5.85	557.9 ± 534.7	/	No significant correlation between retinal changes and HCQ duration. Greater retinal thinning in inner and outer foveal areas in G2 vs. G1. Inferior inner sector showed most significant damage. Nasal inner and temporal inner sectors affected. Superior outer sector showed no abnormalities. Only treatment is discontinuation of HCQ/CQ therapy, though retinopathy may progress after cessation.
Cakir, 2019 [26]	G1a: 44 patients > 5 y HCQ G1b: 30 patients < 5 y HCQ G2: 45 controls	G1a: 1.8 ± 1.1 G1b: 9.6 ± 5.0	G1a: 231.28 ± 215.9 G1b: 794.62 ± 473.2	/	The PR OS length in nasal and temporal region was greater in patients with >5 years of HCQ treatment.
Garrity, 2019 [27]	17 HCQ eyes	11.0 ± 6.5	1611.4 ± 886.2	5.87 ± 1.10	Early HCR that was detected using OCT with normal VF. OCT alterations progress towards OR disruption and VF defects. Early abnormalities were more visible in inferior-temporal parafoveal region, better seen with vertical than horizontal OCT scans.
Allahdina, 2019 [28]	44 eyes with HCR	13.83 ± 7.08	/	5.9 ± 1.8	Progressive retinopathy after drug cessation was detected on autofluorescence imaging. Patients with early HCQ changes can show functional improvement over time, whereas severe retinopathy cannot be reversed.
Pham, 2019 [29]	13 patients with HCR	13.31 ± 5.82	2026.7 ± 1078.1	6.55 ± 2.67	In patients with severe HCR and initial RPE damage, the HCR continued to progress even after medication stopped (20 years off the drug). RPE damage and foveal loss can continue for decades after drug cessation. In early and moderate cases, the progression of HCR stopped after 9 years of medication cessation.
Ruberto, 2018 [30]	33 patients with rheumatic diseases, 28 controls	10.39 ± 8.28	594.5 ± 481.2	3.9 ± 2.6	Significant thinning in outer/inner nasal, outer/inner temporal, and inner superior sectors in HCQ group.
Allahdina, 2018 [31]	G1: 57 HCQ patients G1a: 19 HCR patients G1b: 38 without HCR	G1a: 15.3 ± 7.1 G1b: 14.9 ± 8.3	G1a: 1871 ± 927 G1b: 2036 ± 1141	G1a: 5.61 ± 2.03 G1b: 5.51 ± 1.41	HCR patients showed increased ONL reflectivity on OCT. OCT-MI were elevated significantly in all ETDRS sectors in the retinopathy group compared to the unaffected. OCT-MI reliably detected HCR with high sensitivity and specificity in the inferior quadrant, marking it as a potential biomarker. No significant differences in treatment duration, cumulative dose, or daily dose between affected and unaffected groups.
Ahn, 2018 [32]	G1: 33 patients with HCR G2: 148 HCQ patients without HCR G3: 81 controls	G1: 14.2 ± 4.2 G2: 11.3 ± 5	G1: 1446.9 ± 616.5 G2: 1000.7 ± 526.9	G1: 5.5 ± 1.6 G2: 4.4 ± 1.6	In eyes with HCR, hyporeflective areas with enhanced visibility of deep choroidal vessels on retromode imaging corresponded to OR defects observed on OCT. Retromode imaging using infrared illumination shows promise for visualizing PR and RPE abnormalities and may serve as a useful tool for screening HCR.
Kim, 2017 [33]	G1: 123 HCQ patients G1a: 17 HCR patients	G1: 10.1 G1a: 15.2	G1: 1167.4 G1a: 1866.4	G1: 6.4 G1a: 7.2	Increase in duration of HCQ use, HCQ daily dose, and kidney disease were risk factors in developing HCR. Most defects had a pericentral pattern of HCQ toxicity, with parafoveal and mixed patterns appearing. Retinal toxicity was characterized by OR thinning with disruption of the IS/OS junction on OCT, hyper-autofluorescence on FAF, and patchy VF defects or ring scotomas.
Ahn, 2017 [34]	48 eyes with HCR	13.7 ± 4.5	1482 ± 650	5.8 ± 1.9	Asian patients have a peripheral distribution of PR loss that can cause undetected retinopathy if the PR defect is out of the field of view on current OCT systems. Widefield OCT imaging may identify earlier signs of HCR.
Eo, 2017 [35]	G1: 310 HCQ patients G1a: 9 HCR patients	G1: 6.0 ± 3.4 G1a: 9.1 ± 2.7	G1: 625.5 ± 376.2 G1a: 952.0 ± 519.2	G1: 5.5 ± 1.8 G1a: 5.6 ± 2.6	The frequency of HCR was 5.2% among the patients with HCQ use >5 years. Duration of HCQ > 6 y and total dose >600 g were a significant risk factor. Pericentral and mixed pattern were common in the Korean population.

DLD—drusen-like deposit; FAF—fundus autofluorescence; FT—foveal thickness; G—group; GCIPL—ganglion-cell inner-plexiform layer; HCR—hydroxychloroquine retinopathy; HCQ—hydroxychloroquine; ID—interdigitation; INL—inner nuclear layer; IR—inner retina; IRT—inner retina thickness; IS—inner segment; MI—macular index; MT—macular thickness; mfERG—multifocal electroretinogram; OCT—optical coherence tomography; ONL—outer nuclear layer; OR—outer retina; ORL—outer retinal layers; ORT—outer retinal thickness; OS—outer segment; PR—photoreceptor; PRL—photoreceptor layer; RPE—retinal pigmented epithelium; RT—retinal thickness; SLE—systemic lupus erythematosus; VA—visual acuity; VF—visual field; VIS—visible-light.

**Table 2:** Published studies that analyze HCQ toxicity using OCT focused on ellipsoid zone defects.

Author	Participants	Medication Duration (Years)	Cumulative HCQ Dose (g)	Mean Daily Dose to Real Body Weight (mg/kg)	Key Findings
He, 2024 [36]	120 HCQ eyes	6.4 ± 4.4	821.6 ± 630.3	5.9 ± 2.5	HCR was present in 42 eyes (35.0%). A pericentral pattern was observed in all cases of early HCR. Initially, the damage appeared in the EZ, and then progressed to the RPE. Disruption in these regions was associated with poorer VA.
Yucel Gencoglu, 2024 [37]	61 HCQ patients and 44 controls	7.0 ± 4.9	/	2.81 ± 0.94	Decrease in RPE reflectivity of the temporal, nasal, parafoveal, and nasal perifoveal, EZ, and ELM, compared to foveal in HCQ patients.
Talcott, 2024 [38]	G1a: 38 HCR eyes G1b: 45 HCQ eyes without HCR G2: 44 controls	G1a: 10.9 ± 5.1 G1b: 10.8 ± 3.3	G1a: 1520.1 ± 765.3 G1b: 1493.7 ± 504.3	G1a: 5.6 ± 1.8 G1b: 5.1 ± 1.4	Patients with HCR present higher EZ-At Risk biomarker. Eyes without HCR presented no significant difference compared with controls. EZ disruption associated with HCQ dose on body weight in HCR eyes.
Alieldin, 2022 [39]	344 patients for baseline screening, 566 HCQ patients with risk factors	9 ± 5	/	/	Lower prevalence of HCR than previously reported. HCR patients presented perifoveal atrophic changes with EZ loss and possible ORL and PR disruption.
Kim, 2022 [40]	146 HCQ eyes	14.7 ± 7.1	1340.4 ± 707.3	4.9 ± 1.4	HCR presented a parafoveal pattern (18.5%), pericentral pattern (61.0%), and a mixed pattern (20.5%). Inferior temporal sector was the most common parafoveal and pericentral PR damage location. OCT is better in detecting PR damage in parafoveal or pericentral areas compared to FAF. Clock-hour evaluation of HCR proposed for toxicity assessment.
Kalra, 2022 [41]	388 HCQ eyes evaluated at 2 visits (V1 and V2)	V1: 5.8 ± 3.7 V2: 8.8 ± 3.9	V1: 780 ± 556 V2: 1186 ± 592	4.9 ± 1.5	Treatment duration and HCQ cumulative dose are higher in the HCQ toxicity group. VA was worse in eyes with HCR. Partial EZ attenuation of 1.9% or higher was associated with sensitivity of 88% and specificity of 93% for toxicity detection.
Jayakar, 2022 [42]	84 HCQ patients, G1a: 54 without HCR, G1b: 30 with HCR	G1a: 14.5 ± 7.4 G1b: 14.22 ± 6.57	/	/	There was a reduction in VF sensitivities in a parafoveal configuration. Patients with toxicity showed significant thickness decrease in OR. There was a correlation between VF thickness and OCT. EZ loss is a marker of significant PR loss and severe toxicity.
Dabit, 2022 [43]	G1: 634 HCQ patients G1a: 11 HCR patients	/	G1: 472 G1a: 540	G1: 4.5 G1a: 5.7	Lower prevalence of HCR than previously reported. Cases with retinopathy have parafoveal disruption of the EZ, parafoveal thinning of the ONL, and RPE damage on OCT, and paracentral damage on VF. Longer HCQ treatment duration and higher dosage were risk factors for HCR.
Ahn, 2021 [44]	80 HCR eyes	13.3 ± 5.2	1307.1 ± 437.3	5.0 ± 1.8	Increased EZ defect length, reduced fovea–PR defect distance, and new/enlarged RPE defects are signs of retinopathy progression on OCT. After HCQ cessation, 1/3 of incipient pericentral cases showed limited progression, then stabilization/improvement. Moderate and severe pericentral HCR continued to progress. Retinal thinning rate slows in early/moderate stages. 58.7% of pericentral cases had structural and functional progression. Advanced pericentral pattern showed centripetal enlargement of ring-shaped lesions. Vision loss reported only in severe parafoveal or mixed patterns, especially with foveal involvement.
Jauregui, 2020 [45]	88 HCQ eyes	8 ± 6	994.75 ± 798.85	5.19 ± 1.68	Parafoveal OR structures significantly disrupted, with loss of EZ and ID zone and conservation of the foveal area.
Gobbett, 2021 [46]	678 HCQ patients, 333 patients at risk	/	/	/	Lower prevalence of HCR than previously reported. HCR had perifoveal EZ and RPE loss on OCT.
Browning, 2019 [47]	64 HCQ patients	9.8	1431	4.6	HCR patients had different degrees of bilateral EZ loss, RPE loss, and INL loss. Short, obese women are at higher risk of HCR.
Ugwuegbu, 2019 [48]	14 HCR eyes, 14 controls	20.6 ± 15.0	/	5.5 ± 1.6	Reduced ONL/HFL-EZ volume, parafoveal ONL-HFL-EZ thickness, and EZ–RPE thickness in HCQ toxicity group vs. controls. En-face EZ–RPE mapping valuable for assessing HCQ retinal toxicity.
Arndt, 2018 [49]	G1: 354 HCQ patients G1a: 17 HCR patients	G1a: 6.46 ± 6.47	G1a: 870.2 ± 969.5	G1a: 5.36 ± 1.78	C-Scan OCT is better in detecting early signs of toxicity compared to B-scan based on EZ hyporeflectivity.

ELM—external limiting membrane; EZ—ellipsoid zone; FAF—fundus autofluorescence; G—group; HCR—hydroxychloroquine retinopathy; HCQ—hydroxychloroquine; HFL—Henle fiber layer; ID—interdigitation; INL—inner nuclear layer; OCT—optical coherence tomography; ONL—outer nuclear layer; OR–outer retina; ORL—outer retinal layers; PR—photoreceptor; RPE—retinal pigmented epithelium; V–visits; VA—visual acuity; VF—visual field.

**Table 3:** Published studies that analyze HCQ toxicity using OCT focused on IR alterations.

Author	Participants	Medication Duration (Years)	Cumulative HCQ Dose (g)	Mean Daily Dose to Real Body Weight (mg/kg)	Key Findings
Jung, 2024 [50]	G1: 76 HCQ eyes, G1a: 38 eyes < 5 y HCQ, G1b: 38 eyes > 5 y HCQ, G2: 36 controls	G1: 8.60 ± 6.09, G1a: 4.05 ± 1.14 G1b: 13.42 ± 5.48	/	/	No significant difference in macular GCL thickness between groups. mfERG can detect early subtle electrophysiological changes in HCQ patients.
Sonalcan, 2024 [51]	G1: 60 HCQ eyes > 5 y HCQ, G2: 62 HCQ eyes < 5 y HCQ, 28 patients with RA, SLE, SJS	G1: 8.77 ± 3.01 G2: 2.67 ± 1.09	G1: 639.97 ± 217.52 G2: 194.98 ± 79.57	/	Mean and temporal RNFL thickness significantly lower in HCQ users. FT unchanged between patients and controls. Mean and sectoral GCIPL thickness reduced in HCQ group. Combined functional and structural tests recommended for early HCQ toxicity detection. No intergroup differences among HCQ-treated patients.
Mimier Janczak, 2023 [52]	57 SLE eyes, 56 controls	8.48 ± 7.50	391.23 ± 425.52	/	OCT did not detect subtle early retinal changes. No OCT differences in thickness between SLE and controls regarding retina, RNFL, GCL, and GCIPL. Negative correlation between disease duration/age and inferior RNFL thickness. No correlation between HCQ therapy length and retinal parameters. Lower VA in SLE patients vs. controls. No correlation between RT and laboratory findings.
Agcayazi, 2023 [53]	80 HCQ eyes, 40 controls	6.4 ± 5.1	504.88 ± 387.4	/	No significant differences in MT and parafoveal GCL thickness. Perifoveal GCL thinner in superior nasal, superior temporal, inferior nasal and inferior temporal quadrants in HCQ users. RNFL and GCL thinning useful for evaluating HCQ toxic maculopathy. Thinning correlates with HCQ intake duration. Early pathology involves RGC damage. Later stages show PR and RPE degeneration. Lipofuscin accumulation in RPE leads to toxicity via impaired lysosomal function.
Kim, 2023 [54]	G1: 66 HCQ patients without HCR G2: 66 HCR patients with retinopathy	G1: 9.4 ± 7.7 G2: 15.6 ± 6.9	G1: 797.2 ± 685.5 G2: 1351.7 ± 701.7	G1: 4.5 ± 1.3 G2: 4.7 ± 1.4	Macular GCL and RNFL thinning observed in HCR. Degenerative changes may appear in IRL with disease progression. Retinal toxicity mainly affects the ORL.
Mondal, 2022 [55]	19 HCQ patients and 37 controls	8.36 ± 7.9	16.04 ± 16.72	5.04 ± 1.36	Long-term HCQ treatment causes damage to the RPE, OR, and IR neurons. RGC loss in the perifoveal region is due to typical bull’s-eye appearance of the macula.
Membreno, 2023 [56]	85 HCQ patients, G0: 55 with no signs of toxicity, G1–4: 30 grouped by HCR severity.	G0: 14.5 ± 7.4, G1: 16.7 ± 9.4 G2: 12.2 ± 3.9 G3: 12.0 ± 5.4 G4: 16.7 ± 7.7	/	G0: 5.1 ± 1.6 G1: 4.8 ± 1.8 G2: 5.5 ± 2.1 G3: 6.0 ± 1.5 G4: 6.5 ± 1.8	HCR group had thinner ORT and IRT in nearly all ETDRS subfields. MI values increased across all ETDRS subfields. IR thinning absent only in ETDRS 1. Quantitative OCT metrics differentiate healthy eyes from severe HCR types. OR thinning indicates early/mild HCR. IR thinning associated with severe/advanced stages.
Aydin Kurna, 2022 [57]	G1: 81 patients > 6 months HCQ, G1a > 60 months, G1b < 60 months, G2: 34 rheumatological patients without HCQ, G3: 30 controls	G1a: 8.41 ± 3.91 G1b: 4.17 ± 1.2	G1a: 843.37 ± 489.38 G1b: 208.63 ± 135.01	/	No significant VF differences between G1a and G1b. VF scores significantly worse in G1 vs. G2 and G3. Reduced MT in inner and outer nasal quadrants in G1 compared to the others. Thinner GCL in superior, inferior, inner nasal inferior, and outer temporal superior/inferior regions in G1 compared to the others.
Godinho, 2021 [58]	144 HCQ eyes evaluated at 2 visits (V1 and V2)	V1: 4 V2: 7.19 ± 1.55	V1: 730 V2: 1144.3 ± 229.3	4.89 ± 2.01	Decreased foveolar and paracentral full retina, INL, and GCL thickness. Negative correlation between HCQ treatment duration and foveal GCL thickness. Correlation between age and foveal IRL thickness. IRL thinner after HCQ use.
Martin-Iglesias, 2019 [59]	195 HCQ eyes at 2 visits (V1 and V2)	V1: 5.75 V2: 11.08	V1: 398.5 V2: 774	V1: 3.22 V2: 3.12	No HCR identified over 5-year follow-up. Significant reduction in average macular and GCL thickness in both eyes at 5 years. Confirms long-term HCQ safety at doses < 5 mg/kg/day.
Gil, 2019 [60]	G1: 93 HCQ eyes G1a: 25 eyes < 5 y HCQ G1b: 68 eyes > 5 y HCQ	G1: 8.39 ± 5.09 G1a: 2.96 ± 1.17 G1b: 10.38 ± 4.48	G1: 1020.2 ± 675 G1a: 382.08 ± 167.71 G1b: 1254.81 ± 638.55	G1: 5.09 ± 1.53 G1a: 5.31 ± 1.21 G1b: 5.01 ± 1.63	Long-term HCQ treatment associated with thinner INL. Parafoveal GCL changes observed in patients with high cumulative HCQ dose.
Conigliaro, 2018 [61]	G1: 30 SLE, HCQ patients, G2: 24 SS HCQ patients, G3: 76 controls	G1: 52.3 ± 55.5 G2: 49.1 ± 73.1	G1: 549.7 ± 479.8 G2: 555.3 ± 862.2	/	The SjS patient group showed reduced posterior pole RT, compared to SLE and control group. SLE measurements of pRNFL thickness revealed a reduction in thickness in SS patients in the temporal-inferior and the nasal-inferior sectors compared with HC and SLE patients.
Lee, 2018 [62]	G1: 77 HCQ patients G2: 20 controls	5.3 ± 3.2	528.1 ± 344.1	5.64 ± 1.8	Only temporal pRNFL thinning observed in HCR patients vs. controls. RNFL thickness not reliable for detecting HCR. No correlation between treatment duration, cumulative dose, and RNFL thickness.
Bulut, 2017 [63]	G1: 92 HCQ eyes G2: 80 controls	4.84 ± 3.19	543.6 ± 340.8	/	Thinner GCIPL in superior, superonazal, inferonasal, infratemporal, and superotemporal segments in G1 vs. G2. No difference in peripapillary RNFL thickness between groups. No correlation between cumulative HCQ dose, treatment duration, and average RNFL. GCIPL thickness measurement is an important screening tool for HCR.
Telek, 2017 [64]	G1: 35 eyes of SLE patients with HCQ G2: 40 eyes of RA patients with HCQ G3: 20 controls	G1: 0.58 ± 0.43 G2: 0.63 ± 0.5	/	/	HCQ toxicity was found more in RA patients than in SLE patients. There were statistically significant central foveal thickness differences between eyes with and without IS/OS junction irregularities and between eyes with/without IS/OS junction irregularities and controls. There were no RNFL differences between groups.
Kan, 2017 [65]	90 HCQ eyes > 5 y treatment, 90 controls	6.66	/	/	Patients with HCQ treatment showed significant thinning of the GCIPL, without clinically evident HCQ retinopathy and without VF abnormalities. The macular GCIPL evaluation can be an early detector of HCR.

G—group; GCIPL—ganglion-cell inner-plexiform layer; GCL—ganglion cell layer; HCR—hydroxychloroquine retinopathy; HCQ—hydroxychloroquine; INL—inner nuclear layer; IR—inner retina; IRL—inner retinal layers; IRT—inner retina thickness; IS/OS—inner segment/outer segment; mfERG—multifocal electroretinogram; MI—macular integrity; MT—macular thickness; OCT—optical coherence tomography; OR—outer retina; ORL—outer retinal layers; ORT—outer retinal thickness; PR—photoreceptor; RA—rheumatoid arthritis; RGC—retinal ganglion cell; pRNFL—peripapillary retinal nerve fiber layer; RNFL—retinal nerve fiber layer; RPE—retinal pigmented epithelium; RT—retinal thickness; SJS—Sjögren’s syndrome; SLE—systemic lupus erythematosus; V—visits; VA—visual acuity; VF—visual field.

**Table 4:** Published studies that analyze HCQ toxicity using OCT focused on choroidal alterations.

Author	Participants	Medication Duration (Years)	Cumulative HCQ Dose (g)	Mean Daily Dose to Real Body Weight (mg/kg)	Key Findings
Arias-Peso, 2024 [66]	G1: 32 eyes from <5 y HCQ G2: 44 eyes from >5 y HCQ G3: 46 control	G1: 2.84 ± 1.66 G2: 8.74 ± 4.15	G1: 243.79 ± 162.17 G2: 673.03 ± 362.47	G1: 3.61 ± 1.35 G2: 3.29 ± 1.65	Patients with <5 y HCQ treatment have a thicker choroid, compared to controls. The evolution in central, nasal, and superior sectors was thicker in G1 compared to G3. In the inner superior and outer inferior sectors, CT was thicker in G1 compared to G2. Thinner CT in temporal and inferior sectors was related with HCQ. There were positive correlations between disease activity and HCQ dose. Higher doses of HCQ can control increased disease activity.
Hasan, 2023 [67]	276 HCQ eyes	2.81	215.6	4.5 ± 1.6	There is a relation between increased cumulative dose of HCQ and decreased choroidal volume. The first structures affected in HCR are the PR and the RPE. Decreased vascular and vascularity was associated with HCQ exposure. Choroidal vascular parameters were significantly lower in the study group compared with controls.
Halouani, 2023 [68]	G1: 48 HCQ eyes G1a: 22 eyes mild HCR G1b: 26 eyes HCR G2: 34 controls	G1a: 4.65 ± 3.27 G1b: 13.22 ± 8.86	G1a: 592.4 ± 481.27 G1b: 1825 ± 1026.98	G1a: 4.95 ± 1.31 G1b: 6.35 ± 1.94	The subfoveal mean parameters were lower in advanced HCR compared to the healthy controls. Patients with HCR presented choroidal impairment.
Ru, 2023 [69]	45 JSLE HCQ patients, 50 controls	2.25	188.5	/	Lower CT were found in JSLE compared to healthy controls. HCQ treatment duration and cumulative dose were not corelated with CT. The CT in the macular temporal and subfoveal regions was negatively correlated with IL-6 and IL-10.
Kukan, 2022 [70]	G1: 16 HCQ patients with DLD G2: 16 HCQ patients without DLD	G1: 7.4 G2: 5.3	G1: 567.6 G2: 486.6	G1: 3.8 G2: 4.3	Lower values of the CVI in the patients with DLD compared to the control group. There was no difference between the DLD and without-DLD patients regarding CVI. Eyes with DLD showed greater GCL thickness than the patients without DLD. Eyes with DLD showed thinning in the ORL and PRL compared to the eyes without DLD. DLD can be a factor of disease activity. SLE patients with DLD seem to be in a more active state of disease as compared to patients without such lesions.
Polat, 2022 [71]	G1a: 29 patients < 5 y HCQ; G1b: 20 patients > 5 y HCQ; G2: 39 controls	G1a: 2.01 ± 1.23 G1b: 8.27 ± 4.02	G1a: 248.4 ± 181.6 G1b: 896 ± 480.7	/	HCQ patients: decreased inferior and nasal parafoveal RNFL, and temporal parafoveal GCL and IPL thickness. Increased temporal parafoveal RPE thickness. Reduced subfoveal CT in HCQ group vs. controls. Patients with >5 years of HCQ use show decreased temporal CT vs. controls. Subfoveal and parafoveal CT changes may occur without evident OCT retinal toxicity.
Ahn, 2019 [72]	40 HCR eyes	12.8 ± 3.4	1.430 ± 449	6.1 ± 1.4	Patients showed progression of the retinopathy after drug cessation. Choroidal involvement was found in eyes with severe retinopathy.
Ahn, 2017 [73]	146 HCQ patients, G1: with HCR, G2: without HCR	G1: 12.5 ± 3.7 G2: 7.9 ± 5.5	G1: 1372 ± 450 G2: 780 ± 580	G1: 6.2 ± 1.5 G2: 5.1 ± 1.7	Eyes with HCR showed significantly thinner CT at all measured locations, except at the temporal area 1.5 mm from the fovea. CCT was significantly reduced at all choroidal locations in eyes with HCR compared with eyes without retinopathy. Total CT and CCT at each choroidal location were significantly different between eyes with and without OR defects, including disruption, discontinuity, or attenuation of the EZ, ID zone, or RPE lines, and areas with OR defects demonstrating thinner CT compared with areas without such defects. HCR severity was significantly associated with reduced CT. Subfoveal CCT showed a significant positive correlation with HCQ duration and cumulative HCQ dose adjusted for body weight.
Karti, 2017 [74]	G1: 30 RA patients G2: 30 controls	/	/	/	The subfoveal CT was significantly lower before compared to after treatment, and also lower in G1 compared to G2. CT differences in other locations did not reach statistical significance.

CCT—choriocapillaris thickness; CT—choroidal thickness; CVI—choroidal vascular index; DLD—drusen-like deposits; EZ—ellipsoid zone; G—group; GCL—ganglion cell layer; HCR—hydroxychloroquine retinopathy; HCQ—hydroxychloroquine; ID—interdigitation; IL—interleukin; IPL—inner plexiform layer; JSLE—juvenile systemic lupus erythematosus; OCT—optical coherence tomography; OR—outer retina; PR—photoreceptor; PRL—photoreceptor layer; RNFL—retinal nerve fiber layer; RPE—retinal pigmented epithelium; SLE—systemic lupus erythematosus.

**Table 5:** Published studies that analyze HCQ toxicity focused on OCT-Angiography alterations.

Author	Participants	Medication Duration (Years)	Cumulative HCQ Dose (g)	Mean Daily Dose to Real Body Weight (mg/kg)	Key Findings
Li, 2025 [76]	40 HCQ eyes, 40 controls	6.35 ± 5.37	403.32 ± 220.21	4.68 ± 2.10	Higher inter-eye asymmetry of image VD and perifoveal SVP VD was observed in SLE eyes treated with HCQ compared with the control group. Macular microvascular density was reduced in the HCQ eyes. Optic nerve head VD head did not show any changes. HCQ toxicity is more likely to result in reduced macular VD in the macula. Inter-eye VD asymmetry could be used for the screening of HCQ retinal toxicity.
Bartol-Puyal, 2024 [77]	G1a: 41 HCQ eyes < 10 y SLE, G1b: 31 HCQ eyes > 10 y SLE, G2: 45 controls	G1a: 5.06 ± 2.91 G1b: 7.46 ± 3.88	G1a: 407.50 ± 270.58 G1b: 560.50 ± 334	G1a: 3.46 ± 1.38 G1b: 3.34 ± 0.92	No statistically significant difference between OCT structural and OCT-A VD parameters in patients with SLE.
Araujo, 2024 [78]	132 HCQ eyes	5	396	3.5	4 patients developed subclinical HCQ retinal toxicity without visual acuity impairment. 24% patients have abnormal results in en-face, OCT, and OCTA. In detecting subclinical retinal toxicity, mfERG is more specific than VF. 10 patients had normal results on OCT and VF but abnormal on en-face, OCT, and OCTA
Leclaire, 2024 [79]	24 HCQ eyes	7.2	856.2	/	SLE patients are predisposed to retinal VD reduction, regardless of absent clinical disease activity, due to the physiopathology of the illness itself and not the HCQ therapy.
Zhao, 2024 [80]	43 HCQ patients	3.75	384	5.66 ± 1.66	No correlation between HCR and HCQ blood concentration. Patients with >5 years HCQ treatment had an increased FAZ perimeter compared to patients with < 5 years HCQ treatment. Patients with <5 mg/kg/day had enhanced foveal thickness, compared to patients with >5 mg/kg/day. HCQ treatment duration and cumulative dose is corelated with FAZ. Patients with increased HCQ cumulative doses have decreased foveal VD in SVP. Patients with increased HCQ treatment duration and increased cumulative dose have decreased foveal VD in DVP. Advanced age, large daily doses, and higher cumulative doses were associated with macular area changes.
Liu, 2024 [81]	135 HCQ patients	4	/	4.8	The HCQ cumulative dose and treatment duration was positively correlated with FAZ area and perimeter. There was no correlation between HCQ blood concentration and FAZ area, FAZ perimeter, SVP density, or RT. High HCQ blood concentration level can be a protective factor regarding SLE disease activity. HCQ blood concentration was negatively correlated with IgG, ESR, CRP, and SLED Ai, and positively correlated with HCQ daily doses. There was no correlation between HCQ blood concentration and cumulative dose of HCQ and between HCQ blood concentration and blood parameters. HCQ blood concentration might be associated with SLE disease activity.
Vasilijevic, 2023 [82]	G1: 31 patients with autoimmune disorders; G2: 36 HCQ patients < 5 y; G3: 29 HCQ patients > 5 y.	G2: 2.92 ± 1.53 G3: 10.59 ± 4.63	/	/	Decreased VD in the superficial parafoveal zone in the high-risk group with significantly enlarged FAZ. Superficial VD parameters were modified due to duration of drug use. VF mean deveiation parameters are modified due to disease duration. FT was normal in all 3 groups. HCQ patients with >5 y of treatment appear to have a significant loss of VD in the parafoveal and perifoveal regions, and FAZ area is significantly increased compared to low-risk patients and controls. Drug duration use had a significant negative correlation with VD parameters, and a positive significant correlation with FAZ and OR flow rate parameters. No correlation between disease duration and other parameters.
Esser, 2023 [83]	G1a: 21 HCQ eyes > 5 y, G1b: 9 HCQ eyes < 5 y, 30 controls	G1a: 9.42 ± 4.62 G1b: 3.2 ± 1.69	G1a: 937 ± 599 G1b: 350 ± 242	/	There were no statistically significant VD differences between the study group and the control group. There was no statistically significant dependency between HCQ treatment duration, cumulative HCQ dose, and VD or RT data. The whole en-face RT was significantly reduced in the high-risk group. There was a significantly reduced VD in the superior quadrant of SVP in the high-risk group and a non-significant trend in the parafoveal area of the SVP. VD analysis using OCT-A is not suitable in detecting early signs of HCR in patients with RA.
Liu, 2023 [84]	G1: 10 RA patients without CQ G2: 10 RA CQ patients G3: 10 controls	8.4 ± 1.9	/	/	Patients treated with CQ show reduced VD compared with the RA group that had not been treated with CQ. The patients in RA group had lower VD compared to the control group. The RA group has a decrease in both STMI and SMIR compared to the control group. The conjunctival VD was significantly low in the CQ group compared to the RA group, and decreased in the RA group compared to controls.
Yu, 2023 [85]	G1: 12 SJS patients without HCQ G2: 12 HCQ patients G3: 12 controls	5.5 ± 0.8	/	/	SJS patients have a lower retinal VD compared to the control group. The HCQ group has a decrease in retinal VD. Decreased VA in the HCQ group compared to the SJS group, and also in the SJS group compared to controls. Decreased STMI, SMIR SMAR, DTMI, and DMIR in HCQ group compared to SJS. VD measured by OCT is a promising biomarker than can indicate stages of disease progression. VD decreased further after HCQ treatment, demonstrating that HCQ may contribute significantly to the microvascular alteration in SJS.
Subasi, 2022 [86]	G1a: 44 SLE eyes > 5 y HCQ G1b: 16 SLE eyes < 5 y HCQ G2: 60 controls	/	G1a: 901.22 ± 445.44 G1b: 368.43 ± 195.66	/	Significant decrease in SVP VD including whole image, perifovea, perifovea nasal, inferior, and superior sectors in G1a compared to G1b. SLE duration was longer in G1a compared to G1b. No difference between SLEDAI-2K and SLICC SDI scores. Negative correlation between SVP VD and HCQ cumulative dose in G1a but not in G1b. Significant negative correlation between whole-image DVP VD and cumulative dose in G1a.
Halouani, 2022 [87]	G1: 76 HCQ eyes G1a: 62 HCQ eyes without HCR G1b: 14 HCR HCQ eyes G2: 60 controls	G1a: 9.90 ± 5.38 G1b: 15.85 ± 9.04	G1a: 862.67 ± 592.72 G1b: 2294.29 ± 1274.63	G1a: 5.56 ± 1.09 G1b: 5.75 ± 0.77	G1b eyes had a significantly lower BCVA compared to G1a. G1b eyes had a significantly higher FD percentage and lower FD number. Total FD area was higher in G1b compared to G2. The average size of FD was larger in G1b compared to G1a, with no difference between the control group and G1a. Patients with HCR have a significantly higher CC hypoperfusion and higher FD density percentage
Forte, 2021 [88]	20 HCQ eyes, 36 controls	10.03 ± 3.25	937.59 ± 332.37	4.46 ± 1.47	Despite normal BCVA, FAF, mfERG, VF, and OCT, OCTA showed VD reduction in the central, nasal, and temporal subfields of DVP and in central subfields of CC and increased FAZ in the 3 capillary plexi. Reduced central FT in patients with >5 y of treatment.
Akhlaghi, 2021 [89]	61 HCQ patients (40 eyes had abnormal mfERG and 21 had normal mfERG)	normal mfERG 13.23 ± 5.83, abnormal mfERG 13.15 ± 5.63	normal mfERG- 978.20 ± 523.20, abnormal mfERG 1034.77 ± 590.09	/	There were no significantly differences when evaluating the VD in the SVP between normal mfERG and abnormal mfERG groups. In the RA patients, VD in SVP in perifoveal region was lower in abnormal mfERG group compared to normal mfERG group. VD in DVP in whole-image, superior hemi, inferior hemi, and perifoveal areas was significantly lower in the abnormal mfERG group compared to normal mfERG group in the RA patients. No changes were found in the SLE group. There were no statistically significant changes when evaluating FAZ, flow area of OR, and CC flow area between the abnormal/normal mfERG groups. In RA patients with abnormal mfERG, the mean flow area of CC was significantly lower than in RA with normal mfERG group. The OCT changes are corelated with treatment duration and cumulative dose of HCQ.
Cinar, 2021 [90]	28 HCQ eyes, 28 controls	5.25 ± 0.93	593.714 ± 450	/	HCQ eyes had smaller flow areas in the SVP, DVP, and CCP, than the non-HCQ group. HCQ eyes had macular flow areas that are significantly smaller compared to controls. An enlarged FAZ area was observed in HCQ group compared to the control group. Decreased central FT and SFCT in HCQ group compared to controls. All changes were corelated with cumulative HCQ doses, but not with disease duration. Patients in the high-risk group and with >6.5 mg/kg HCQ daily have decreased VD in foveal, parafoveal, temporal superior, nasal inferior, SVP, and DVP. Low-risk patients have lower superficial and deep VD compared to controls.
Tarakcioglu, 2020 [91]	G1: 70 HCQ eyes G1a: 41 eyes > 5 y HCQ G1b: 29 eyes < 5 y HCQ G2: 32 control eyes	G1: 6.11 ± 3.71 G1a: 2.47 ± 1.2 G1b: 8.52 ± 2.7	/	/	Lower thickness parameters for superficial and deep retina in the HCQ group compared to the control group. Lower SVP VD in >5 years HCQ treatment group.
Lenfant, 2020 [92]	G1: 23 HCR patients G2: 547 HCQ patients without HCR	G1: 16.2 G2: 6.6	G1: 2338 G2: 884	/	Risk factors for developing HCR: age, height, creatinine clearance, hemoglobin concentration, duration of HCQ intake, higher cumulative dose, and geographic origin. HCQ blood level was not a significant risk factor for developing HCR. Low HCQ level is a predictor of SLE exacerbation. High HCQ levels are associated with cutaneous hyperpigmentation lesions and retinopathy. Patients from West Indies or Sub-Saharan Africa have a higher risk of developing HCR.
Mihailovic, 2020 [93]	19 HCQ eyes without retinopathy; 19 healthy controls	5.76 ± 5.18	819 ± 773	/	Reduced VD in the en-face SVP in the high-risk group and low-risk group compared to controls. In the SLE patients, there was lower VD of CC and larger FAZ area. HCQ might have a protective role on retinal microvasculature on SLE patients. SLE can decrease retinal VD.
Ozek, 2019 [94]	G1: 24 eyes > 5 y HCQ G2: 16 eyes < 5 y HCQ G3: 20 controls	G1: 123.9 ± 21.5 G2: 22.81 ± 10.5	G1: 520.34 ± 112.71 G2: 330.12 ± 138.56	/	Statistically significant decrease in DVP VD in the group with higher HCQ cumulative dose compared to controls in the temporal and hemi-inferior sectors. Statistically significant decrease in RT corresponding to SVP in inferior, temporal, and DVP hemi inferior region, the lowest in the >5 y HCQ intake group. No VF significant differences between the groups.
Conigliaro, 2019 [95]	52 HCQ eyes, 40 controls	15.1 ± 7.7	738.8 ± 486.8	/	There was a lower VD on superficial whole en-face, parafoveal, and foveal seen in the SLE eyes, compared to controls. Parafoveal VD and parafoveal thickness were reduced in patients with SLE nephritis, compared to controls. Density of the superficial whole en-face, foveal, parafoveal, and deep whole en-face and parafoveal were negatively corelated with SLE duration. Superficial and deep parafoveal VD was positively corelated with HCQ cumulative dose.
Goker, 2019 [96]	40 HCQ eyes, 40 controls	5.85 ± 0.84	/	3.08	Significantly larger FAZ perimeter and area in HCQ group. Foveal VD in the SVP and DVP was significantly lower in the HCQ group.
Bulut, 2018 [97]	G1: 30 HCQ eyes > 5 y G2: 30 HCQ eyes < 5 y	G1: 7.73 ± 3.57 G2: 1.68 ± 1.36	G1: 746.73 ± 446.52 G2: 137.67 ± 104.56	G1: 3.60 ± 1.03 G2: 3.50 ± 0.85	Statistically significant decrease in VD of the SVP, DVP, and whole, foveal, and parafoveal zones in G1 compared to G2. Wider FAZ at both superficial and deep levels in G1 compared to G2. Decrease in retinal flow rates in G1 compared to G2. No difference regarding MT between groups. CT thinner in G1 compared to G2. Regarding VF, mean deficit was significantly increased in G1 compared to G2. Cumulative dose and treatment duration were positively corelated with FAZ parameter, and negative corelated with vascular density, flow rate, and CT parameters.

BCVA—best corrected visual acuity; CC—choriocapillaris; CCP—choriocapillaris plexus; CQ—chloroquine; CRP—C reactive protein; CT—choroidal thickness; DAI—Disease Activity Index; DMIR—deep macular ischemia ratio; DTMI—deep total microvascular index; DVP—deep vascular plexus; ESR—erythrocyte sedimentation rate; FAF—fundus autofluorescence; FAZ—foveal avascular zone; FT—foveal thickness; G—group; HCR—hydroxychloroquine retinopathy; HCQ—hydroxychloroquine; Ig—immunoglobulin; mfERG—multifocal electroretinogram; MT—macular thickness; OCT—optical coherence tomography; OCT-A—optical coherence tomography angiography; OR—outer retina; RA—rheumatoid arthritis; RT—retinal thickness; SVP—superficial vascular plexus; SFCT—subfoveal choroidal thickness; SJS—Sjögren’s syndrome; SLE—systemic lupus erythematosus; SLICC SDI—Systemic Lupus International Collaborating Clinics — Systemic Damage Index; SMAR—superficial macular area ratio; SMIR—superficial macular ischemia ratio; STMI—superficial total microvascular index; VA—visual acuity; VD—vascular density; VF—visual field.

## Data Availability

The original contributions presented in this study are included in the article. Further inquiries can be directed to the corresponding author.

## Abbreviations

The following abbreviations are used in this manuscript:

BCVA	Best corrected visual acuity
CC	Choriocapillaris
CCP	Choriocapillaris plexus
CCT	Choriocapillaris thickness
CMT	Central macula thickness
CQ	Chloroquine
CRP	C Reactive Protein
CT	Choroidal thickness
CVI	Choroidal vascular index
DAI	Disease activity index
DLD	Drusen-like deposit
DMIR	Deep macular ischemia ratio
DTMI	Deep total microvascular index
DVP	Deep vascular plexus
ELM	External limiting membrane
ESR	Erythrocyte sedimentation rate
EZ	Ellipsoid zone
FAF	Fundus autofluorescence
FAZ	Foveal avascular zone
FFA	Fundus fluorescein angiography
FD	Flow-related deficits
FT	Foveal thickness
G	Group
GCL	Ganglion cell layer
GCIPL	Ganglion cell inner-plexiform layer
HCR	Hydroxychloroquine retinopathy
HCQ	Hydroxychloroquine
HFL	Henle fiber layer
ID	Interdigitation
IL	Interleukin
Ig	Immunoglobulin
INL	Inner nuclear layer
IPL	Inner plexiform layer
IR	Inner retina
IRL	Inner retinal layers
IRT	Inner retina thickness
IS	Inner segment
JSLE	Juvenile systemic lupus erythematosus
LCA	Luminal choroidal area
mfERG	Multifocal electroretinogram
MI	Macular integrity
MT	Macular thickness
NIR	Near-infrared reflectance
NIR-AF	Near-infrared autofluorescence
ONL	Outer nuclear layer
OCT	Optical coherence tomography
OCT-A	Optical coherence tomography angiography
OR	Outer retina
ORL	Outer retinal layers
ORT	Outer retina thickness
OS	Outer segment
PR	Photoreceptor
PRL	Photoreceptor layer
pRNFL	Peripapillary retinal nerve fiber layer
QOL	Quality of life
RA	Rheumatoid arthritis
RGC	Retinal ganglion cell
RPC	Radial peripapillary capillaries
RPE	Retinal pigmented epithelium
RNFL	Retinal nerve fiber layer
RT	Retinal thickness
SCA	Stromal choroidal area
SFCT	Subfoveal choroidal thickness
SJS	Sjögren’s syndrome
SLE	Systemic lupus erythematosus
SLICC-SDI	Systemic Lupus International Collaborating Clinics–Systemic Damage Index
SMAR	Superficial macular area ratio
SMIR	Superficial macular ischemia ratio
SS	Systemic sclerosis
STMI	Superficial total microvascular index
SVP	Superficial vascular plexus
SW-AF	Short-wave autofluorescence
TCA	Total choroidal area
V	Visit
VA	Visual acuity
VD	Vessel density
VF	Visual field
VIS	Visible-light
VRQOL	Vision-related quality of life
